# P-2214. The Microbiome of Community-Dwelling Adults and Risk Factors for Colonization with Antibiotic-Resistant Bacteria in Chile

**DOI:** 10.1093/ofid/ofae631.2368

**Published:** 2025-01-29

**Authors:** Araos Rafael, Juan Ugalde, Alejandro Jara, Felipe Sanchez, Lina M Rivas, Jose M Munita, Erika M C D’Agata

**Affiliations:** Universidad del Desarrollo, Santiago, Region Metropolitana, Chile; Universidad Andrés Bello, Santiago, Region Metropolitana, Chile; Pontificia Universidad Católica de Chile, Santiago, Region Metropolitana, Chile; Pontificia Universidad Católica de Chile, Santiago, Region Metropolitana, Chile; Genomics & Resistant Microbes (GeRM), Instituto de Ciencias e Innovación en Medicina, Facultad de Medicina Clínica Alemana, Universidad del Desarrollo, Chile; Millennium Initiative for Collaborative Research on Bacterial Resistance (MICROB-R), Santiago, Region Metropolitana, Chile; Instituto de Ciencias e Innovación en Medicina, Santiago, Region Metropolitana, Chile; Brown University, Providence, Rhode Island

## Abstract

**Background:**

Intestinal colonization due to antibiotic-resistant bacteria (ARB) often precedes episodes of infection and fosters the spread of antibiotic resistance. This study explores the clinical, epidemiological, and microbiome factors associated with ARB colonization in Chilean adults.

**Methods:**

A cross-sectional study of 357 community-dwelling individuals from a community in central Chile was conducted between December 2018 - May 2019, using stool samples and health questionnaires. Stool samples were cultured on selective media, followed by identification with MALDI-TOF and disc diffusion susceptibility method. Fecal microbiome assessments were conducted using 16S rRNA gene sequencing performed on Illumina MiSeq. ARB colonization was defined as phenotypic resistance of recovered isolates to extended-spectrum cephalosporins, fluoroquinolones, and/or carbapenems.

Associations between colonization and clinical and epidemiological factors were analyzed through classification trees and logistic regression, while diversity and composition of microbiomes were compared between participants based on colonization status.

**Results:**

No exposures were associated with the risk of ARB colonization. The microbiomes of colonized individuals featured Firmicutes, with Bacteroidetes, Actinobacteria, and Proteobacteria also present, paralleling the microbiome profile of the non-colonized individuals. However, ARB-colonized individuals had an overrepresentation of *Escherichia/Shigella*, *Klebsiella*, and *Enterococcus* genera. There were no differences in alpha or beta diversity by ARB colonization status.

**Conclusion:**

The lack of risk factors regarding ARB colonization suggests that such colonization might occur without overt selective pressures. However, the overrepresentation of specific genera in colonized individuals could subtly indicate the influence of unrecognized selective pressures, such as environmental exposure to antibiotic residues. This scenario underscores the complexity of ARB dynamics and the need for further studies to explore potential environmental and indirect factors contributing to ARB colonization in the community setting.

**Disclosures:**

All Authors: No reported disclosures

